# Genetic combining ability of coriander genotypes for agronomic and phytochemical traits in response to contrasting irrigation regimes

**DOI:** 10.1371/journal.pone.0199630

**Published:** 2018-06-28

**Authors:** Amir Gholizadeh, Hamid Dehghani, Mostafa Khodadadi, Patrick J. Gulick

**Affiliations:** 1 Department of Plant Genetics and Breeding, Faculty of Agriculture, Tarbiat Modares University, Tehran, Iran; 2 Department of Biology, Concordia University, Montreal, Quebec, Canada; College of Agricultural Sciences, UNITED STATES

## Abstract

Knowledge of genetic combining ability and gene action would help breeders to choose suitable parents and devise an appropriate breeding strategy for coriander. In the present study, six diverse genotypes of coriander, their 15 F_1_s and 15 F_2_s were evaluated through randomized complete block design with three replications to study genetic combining ability for agronomic and phytochemical traits in coriander. Plants were subjected to well-watered (WW), mild water-deficit stress (MWDS) and severe water-deficit stress (SWDS) irrigation regimes. The results indicate that water-deficit stress decreased all of the measured traits in both the F_1_ and F_2_ generations. General combining ability and specific combining ability effects were highly significant for all of the traits in both the F_1_ and F_2_ generations. Additive gene action was predominant for phonology and fruit yield component traits in all irrigation regimes in both the F_1_ and F_2_ generations. For fatty acid content and total lipid yield, non-additive gene action was predominant in the F_1_ generation while additive gene action was predominant in the F_2_ generation under MWDS and SWDS conditions. The P_4_ parent had the highest general combining ability for fruit yield components in both the F_1_ and F_2_ generations. The P_6_ parent had the highest general combining ability for phenological and phytochemical traits. The P_4_ and P_6_ parents are promising material to develop early flowering and early maturing genotypes coupled with high total lipids in advanced generations of segregation.

## Introduction

Coriander (*Coriandrum sativum* L.) is an annual herb that belongs to the umbelliferous plant family, the Apiaceae. The rapid life cycle of some coriander genotypes allow them to be cultivated in the wide range of geographical areas throughout the world [1]. Fresh and dried leaves and seeds are commonly used as a seasoning and a general food ingredient [2]. Coriander is mainly cultivated for its fruit characteristics that are used for different applications in the food, drug, cosmetic, and perfume industries [3]. Coriander fruit contains oils with a high concentration of monounsaturated fatty acids [4, 5–7]. The oils with different fatty acid compositions are important for human consumption and for industrial uses; oleic, linoleic and petroselinic acids are the main components of fatty acids in coriander. Oils with a high proportion of oleic acid are more stable than other vegetable oils and they are recommended in the diet to reduce the risk of cardiovascular diseases in humans [8]. On the other hand, linoleic acid is preferred by industries when oil hydrogenation is required and it is an essential fatty acid in the human diet. Petroselinic acid can be broken down into adipic (C6) and lauric (C12:0) acids by oxidative cleavage. Adipic acid is used for the manufacture of a wide range of polymers including high-grade engineering plastics. Lauric acid is used as a raw material for soaps, emulsifiers, detergents, and softeners [9].

The development of new crops for the production of industrial oils is an area of significant interest both scientifically and environmentally [4]. The quantity and composition of fatty acids may be affected by growth conditions, including water-deficit stress which can lead to change in the morphology, physiology and biochemistry of plants [10–12]. There are several evidences that water-deficit stress can significantly decrease the fatty acid content and yield in plants such as safflower (*Carthamis tinctorius* L.) [13], sage (*Salvia officinalis* L.) [11], cumin (*Cuminum cyminum* L.) [14] and soybean (*Glycine max* L.) [15]. Therefore, development of drought-tolerant cultivars with a high total lipid yield is an important area of research in medicinal and industrial plants such as coriander.

Drought tolerance is defined as the ability of plants to live, grow and produce yield under water-deficit stress conditions [16]. Several studies have reported that the water-deficit stress can lead to reduced oil content and yield in oil seed and culinary seed crops including coriander [17, 18], dill (*Anethum graveolens* L.) [19], *Plantago ovata* and *Nigella sativa* [20], caraway (*Carum carv*i L.) [21], purple basil (*Ocimum basilicum* L.) [22] and cumin [23]. Yield is a complex quantitative trait that is affected by various phenological and yield component traits, each with its own genetic systems. The component traits can also be used as surrogate traits to assess drought tolerance and to identify genotypes with high yield potential for use in coriander improvement programs. Flowering time and maturity traits are known as the important factors in determining yield, moreover these traits can easily be evaluated by simple observation under field conditions [24]. Amiri-Oghan et al. [25] noted that days to early flowering and late maturity can be used as suitable indicators to screen for high yielding oilseed rape (*Brassica napus* L.) genotypes under water restricted conditions. Khodadadi et al. [26] showed that there was a significant negative correlation between days to flowering and fruit yield in coriander under water-deficit stress and that early flowering, a component of drought escape, enhanced fruit productivity in coriander under water-deficit conditions. Yield components can also be used as indicators for identifying the high yielding genotypes due to their ease of measurement and high heritability. When traits are governed by similar genetic control mechanisms these traits could simultaneously be improved by selection under water-deficit conditions [18].

Knowledge of the extent and nature of the genetic architecture and heritability of the major traits associated with yield and correlations between traits are essential to improve the efficiency of breeding programs [27]. The diallel mating design has been used to quantify the nature of gene action which control traits and also to estimate general combining ability (GCA) and specific combining ability (SCA) of parents and crosses, respectively. Examples of its use in a wide array of crops include those of Gao et al. [28] in *Agaricus bisporus*, Townsend et al. [29] in *Artemisia annua*, Zhang et al. [30] in barley, Khodadadi et al. [18] in coriander, dos Santos et al. [31] in *Theobroma cacao*, Pereira et al. [32] in cacao and Hirut et al. [33] in potato. GCA is defined as the average yield of a parental genotype over relevant hybrids and corresponds to additive genetic effects, while the SCA is defined as the yield of a hybrid that deviates from what would be expected if traits were controlled by additive effects alone, i.e. it represents non-additive genetic effects [34].

There is insufficient knowledge of the genetic control of phenological and yield component characteristics in coriander. The objectives of this study were (1) to quantify heritability and the nature of gene action controlling phonological traits, yield components and total lipid yield traits, and (2) to estimate genetic combining ability of parents and hybrids.

## Materials and methods

### Plant material and growth conditions

The coriander genotypes used to make diallel crosses had been evaluated in a preliminary experiment for drought tolerance by Khodadadi et al. [35]. Parents included the commercial genotype (P_1_), TN-59-353 (P_2_; relatively drought tolerant), TN-59-80 (P_3_; drought susceptible), TN-59-160 (P_4_; drought tolerant and relatively high yielding), TN-59-158 (P_5_; highly drought susceptible) and TN-59-230 (P_6_; highly drought tolerant but low yielding). All six parents were used in half diallel mating design, without reciprocals, to produce 15 F_1_ hybrids in 2014. Seeds of these F_1_ hybrids were used to produce 15 F_2_ generations through self-pollination in isolated conditions. All of the six parents, the F_1_ hybrids and F_2_ populations were evaluated in different irrigation regimes in experiments with a randomized complete block design with three replications in each experiment during growing season of 2016. Tests were carried out at the research field of Tarbiat Modares University in Iran (51° 09 ^ʹ^E longitude and 35° 44^ʹ^ N latitude, at an elevation of 1265 m above sea level). In treatment 1, genotypes were kept well-watered overall (WW). In treatment 2, genotypes were well-watered until the commencement of stem elongation when watering was withdrawn until the end of the flowering stage at which time only one recovery watering was applied (mild water-deficit stress; MWDS). In treatment 3, watering was normal until the commencement of the flowering stage, after which watering was cut off completely (severe water-deficit stress; SWDS). The soil’s physical and chemical characteristics in the experimental field are presented in S1 Table.

### Trait measurements

The traits which were measured included days to flowering (DTF), days to the end of flowering (DTEOF), days to ripening (DTR), umbel number per plant (UNPP), fertile umbel number per plant (FUNPP), fruit number per plant (FNPP), thousand fruit weight (TFW), fatty acid content (FAC) and total lipid yield (TLY). The timing of phenological traits were noted at the time at which 50% of plants in each plot had reached the target phonological stage. Sample size to measure yield components varied with genetic material; FAC and TLY traits were measured in ten plants in each plot for parental genotypes and F_1_ hybrids and in 30 plants in each plot for F_2_ populations.

To measure fatty acid content, two grams of powdered fruit samples of coriander were subjected extraction with a Soxhlet apparatus with 250 ml of petroleum ether for 6 h. Fatty acid content was measured after filtration and solvent evaporation under reduced temperature and pressure [18]. Finally, total lipid yield was estimated by multiplying fatty acid content with fruit yield per plant (g) for each plot.

### Statistical analysis

The datasets were first tested for normality according to the Shapiro-Wilk test method [36]. The analysis of variance for GCA and SCA effects was done according to Griffing’s [37] method 2, model 1 using a SAS program proposed by Zhang et al. [38]. Mean values of the traits in different irrigation regimes were compared using least significant difference (LSD) method. Estimates of $\sigma_{g}^{2}$ (general combining ability variance) and $\sigma_{s}^{2}$ (specific combining ability variance) were computed based on the random-effects model of Griffing’s [37] method. These estimates were used to calculate *σ*_A_^2^ (additive variance), *σ*_D_^2^ (dominance variance), h^2^ (heritability), and the GCA/SCA ratio [38]. The relative importance of variances due to GCA and SCA were computed for the traits using the method proposed by Baker [39] (Eq 1).

$$\mathrm{GCA}/\mathrm{SC}A_{\mathrm{ratio}}=\frac{2\sigma_{g}^{2}}{2\sigma_{g}^{2}+\sigma_{s}^{2}}$$ (1)

The GCA/SCA ratio reflects the degree of trait transmission from parent to the progeny. When the GCA/SCA ratio is closer to one, it shows that additive gene action is largely involved in the inheritance of the trait and it will be well transmitted from the parents to the progenies. Whereas, a GCA/SCA ratio closer to zero shows that non-additive gene action is predominant in the inheritance of the trait. Narrow-sense heritability (h_N_^2^) was computed according to Eq 2 [40].

$$h_{N}{}^{2}=\frac{\sigma_{A}{}^{2}}{\sigma_{A}{}^{2}+\sigma_{D}{}^{2}+\frac{\sigma_{E}{}^{2}}{r}}$$ (2)

where *σ*_E_^2^ and r are the error variance and number of replications, respectively. The genotypic correlation coefficients between traits were calculated according to the formula proposed by Holland [41]. The statistical analysis was carried out using SAS [42] software.

## Results

### Combined analysis of variance of traits

Combined analysis of variance demonstrated that there were significant effects of different irrigation regimes on all of the traits in both the F_1_ hybrids and the F_2_ populations (S2 Table). Genetic differences between F_1_ hybrids and between F_2_ populations were highly significant for all of the studied traits. These results indicate that parents for diallel crosses had been properly selected. Also, genotype × irrigation regime interaction effects were significant for all traits in both F_1_ hybrids and F_2_ populations (S2 Table). The GCA and SCA effects were significant for all traits. Also, GCA × irrigation regime interaction effect was significant for all the traits in both the F_1_ and F_2_ generations. The SCA × irrigation regime interaction effect was significant for all traits in both the F_1_ and F_2_ generations except for TFW trait.

### Effect of water-deficit stress on measured traits

DTF, DTEOF, DTR, UNPP, FUNPP, FNPP, TFW, FAC and TLY were significantly reduced under MWDS and SWDS irrigation regimes compared to the WW irrigation regime (S3 Table).

### Nature of gene action

The GCA and SCA variances were highly significant for all traits in both the F_1_ and F_2_ generations (Tables 1 and 2). The GCA/SCA ratio values were high for phenological traits and relatively high for yield components in all irrigation regimes (Tables 1 and 2). These results indicate that additive gene action was predominant in controlling these traits. For FAC non-additive gene action was predominant in the F_1_ generation, while additive gene effects were important in the F_2_ generation under all irrigation regimes (Tables 1 and 2). In addition, in WW condition, non-additive gene action was predominant for TLY in both the F_1_ and F_2_ generations (Tables 1 and 2). Under MWDS and SWDS irrigation regimes, non-additive gene action was predominant for TLY in the F_1_ generation, while additive gene effects were important in the F_2_ generation (Tables 1 and 2).

**Table 1:** Analysis of variance for combining ability, variance components, heritability and GCA/SCA ratio estimates in the F_1_ generation under different irrigation regimes.

IR	E	DTF	DTEOF	DTR	UNPP	FUNPP	FNPP	TFW	FAC	TLY
**WW**	GCA	1403.63**	1198.78**	753.81**	1077.65**	3741.60**	733667.03**	19.11**	59.34**	16.25**
	SCA	24.55**	23.32**	6.90**	186.70**	341.13**	69891.17**	2.28**	28.44**	26.53**
	Error	2.58	0.95	0.77	40.78	34.09	13083.67	0.25	2.33	0.08
	$\sigma_{g}^{2}$	57.46**	48.98**	31.12**	37.12**	141.69**	27657.33**	0.70**	1.29^ns^	0.03^ns^
	$\sigma_{s}^{2}$	7.32**	7.46**	2.04**	48.64**	102.35**	18935.83**	0.67**	8.70**	0.64**
	$h_{N}^{2}$	0.93	0.93	0.96	0.54	0.71	0.70	0.65	0.19	0.08
	GCA/SCA	0.94	0.93	0.97	0.60	0.73	0.74	0.68	0.23	0.09
**MWDS**	GCA	1585.53**	1169.13**	863.31**	340.37**	40.08**	214715.62**	53.68**	101.93**	2.873**
	SCA	69.52**	73.47**	59.62**	53.11**	4.09*	26472.49**	2.59**	23.03**	0.791**
	Error	1.27	0.86	1.45	13.77	2.05	1120.88	0.50	1.68	0.049
	$\sigma_{g}^{2}$	63.17**	45.65**	33.49**	11.97**	1.50**	7843.46**	2.13**	3.29*	0.006*
	$\sigma_{s}^{2}$	22.75**	24.20**	19.39**	13.11**	0.68*	8450.54**	0.70**	7.12**	0.009**
	$h_{N}^{2}$	0.85	0.79	0.77	0.57	0.69	0.64	0.83	0.43	0.17
	GCA/SCA	0.85	0.79	0.78	0.65	0.82	0.65	0.86	0.48	0.41
**SWDS**	GCA	1952.31**	844.83**	558.82**	64.48**	30.06**	173857.19**	47.58**	76.03**	0.68**
	SCA	73.74**	42.26**	46.20**	16.38*	6.85**	19795.43**	3.08**	22.73**	0.20**
	Error	1.64	1.09	0.97	7.52	2.03	2354.24	0.20	1.94	0.03
	$\sigma_{g}^{2}$	78.27**	33.44**	21.36**	2.00*	0.97**	6419.24**	1.85**	2.22*	0.02*
	$\sigma_{s}^{2}$	24.03**	13.72**	15.08**	2.95*	1.61**	5813.73**	0.96**	6.93**	0.06**
	$h_{N}^{2}$	0.86	0.83	0.74	0.42	0.46	0.66	0.78	0.33	0.31
	GCA/SCA	0.87	0.83	0.74	0.58	0.55	0.69	0.79	0.39	0.41

**, * and ^ns^ indicate significance at the 1% and 5% level of probability and not significant, respectively. General combining ability (GCA), specific combining ability (SCA), variance of general ($\sigma_{g}^{2}$) and specific ($\sigma_{s}^{2}$) combining ability, narrow-sense heritability ($h_{N}^{2}$), GCA/SCA ratio, irrigation regime (IR), estimates (E), well-watered (WW), mild water-deficit stress (MWDS), severe water-deficit stress (SWDS), days to flowering (DTF), days to the end of flowering (DTEOF), days to ripening (DTR), umbel number per plant (UNPP), fertile umbel number per plant (FUNPP), fruit number per plant (FNPP), thousand fruit weight (TFW), fatty acid content (FAC), total lipid yield (TLY).

**Table 2:** Analysis of variance for combining ability, variance components, heritability and GCA/SCA ratio estimates in the F_2_ generation under different irrigation regimes.

IR	E	DTF	DTEOF	DTR	UNPP	FUNPP	FNPP	TFW	FAC	TLY
**WW**	GCA	1567.91**	1308.28**	924.18**	981.17**	3539.76**	543433.19**	15.66**	30.62**	8.30**
	SCA	45.67**	40.58**	7.14**	128.32**	255.71**	29702.02*	1.47**	6.88**	6.08**
	Error	5.40	1.23	0.79	35.17	28.23	13783.29	0.26	2.19	0.05
	$\sigma_{g}^{2}$	63.43**	52.82**	38.21**	35.54**	136.84**	21405.47**	0.59**	0.99**	0.004^ns^
	$\sigma_{s}^{2}$	13.42**	13.12**	2.12**	31.05**	75.83**	5306.24*	0.40**	1.56**	0.08**
	$h_{N}^{2}$	0.87	0.86	0.96	0.59	0.72	0.80	0.66	0.35	0.05
	GCA/SCA	0.90	0.89	0.97	0.70	0.78	0.89	0.75	0.56	0.10
**MWDS**	GCA	1616.58**	1258.57**	842.25**	186.85**	34.39**	184042.28**	45.15**	119.15**	2.147**
	SCA	79.20**	83.79**	62.88**	67.34*	3.37**	14090.57**	1.60**	16.00**	0.448**
	Error	1.35	1.00	1.55	41.28	1.28	1790.59	0.49	1.70	0.071
	$\sigma_{g}^{2}$	64.06**	48.95**	32.47**	4.98*	1.29**	7081.32**	1.81**	4.30**	0.003**
	$\sigma_{s}^{2}$	25.95**	27.60**	20.44**	8.69*	0.70**	4099.99**	0.37**	4.77**	0.003**
	$h_{N}^{2}$	0.79	0.73	0.70	0.31	0.67	0.71	0.86	0.53	0.09
	GCA/SCA	0.83	0.78	0.76	0.53	0.79	0.78	0.91	0.64	0.53
**SWDS**	GCA	1897.50**	928.53**	451.62**	53.19**	20.67**	153701.14**	40.89**	59.48**	0.48**
	SCA	77.60**	52.98**	24.60**	9.59**	3.75**	15254.07**	2.04**	11.56**	0.12**
	Error	2.49	1.70	2.85	3.32	0.71	1811.94	0.23	2.37	0.03
	$\sigma_{g}^{2}$	75.83**	36.48**	17.79**	1.82**	0.71**	5768.63**	1.62**	2.00**	0.02**
	$\sigma_{s}^{2}$	25.03**	17.09**	7.25**	2.09**	1.01**	4480.71**	0.61**	3.06**	0.03**
	$h_{N}^{2}$	0.82	0.76	0.77	0.50	0.48	0.64	0.79	0.41	0.39
	GCA/SCA	0.86	0.81	0.83	0.63	0.58	0.72	0.84	0.57	0.57

**, * and ^ns^ indicate significance at the 1% and 5% level of probability and not significant, respectively. General combining ability (GCA), specific combining ability (SCA), variance of general ($\sigma_{g}^{2}$) and specific ($\sigma_{s}^{2}$) combining ability, narrow-sense heritability ($h_{N}^{2}$), GCA/SCA ratio, irrigation regime (IR), estimates (E), well-watered (WW), mild water-deficit stress (MWDS), severe water-deficit stress (SWDS), days to flowering (DTF), days to the end of flowering (DTEOF), days to ripening (DTR), umbel number per plant (UNPP), fertile umbel number per plant (FUNPP), fruit number per plant (FNPP), thousand fruit weight (TFW), fatty acid content (FAC), total lipid yield (TLY).

### Narrow-sense heritability

Heritability estimates for all traits are presented in Tables 1 and 2. Under WW conditions, narrow-sense heritability estimates covered a wide range of values among the different traits. They were highest for DTR where they were 0.96 in both the F_1_ and F_2_ generations. Hereditability estimates were lowest for TLY; they were 0.08 and 0.05 in the F_1_ and F_2_ generations, respectively. In MWDS, narrow-sense heritability of traits ranged from 0.17 for TLY to 0.85 for DTF in the F_1_ generation and 0.09 for TLY to 0.86 for DTF in F_2_ generation. Also under SWDS, narrow-sense heritability estimates ranged from 0.31 to 0.86 in F_1_ generation and from 0.39 to 0.82 in the F_2_ generation for TLY and DTF, respectively. Moderate to high values of narrow-sense heritability were observed for DTF, DTEOF, DTR, UNPP, FUNPP, FNPP, TFW and FAC traits under all irrigation regimes. Whereas, low values of narrow-sense heritability were obtained for TLY under all irrigation regimes (Tables 1 and 2).

### Genetic combining ability analysis

GCA values of parents in both the F_1_ and F_2_ generations showed that the P_6_ parent was the best general combiner for phenological traits that enable plants to reach early ripening in all irrigation regimes; it had the largest negative GCA value for days to flowering (Table 3). In the case of UNPP, FUNPP and FNPP traits, the P_4_ parent was the best general combiner in both the F_1_ and F_2_ generations in all irrigation regimes (Table 3). Also, the P_6_ appeared as the best general combiner for TFW and FAC in all irrigation regimes in both the F_1_ and F_2_ generations. In the case of TLY, the P_4_ parent was the best general combiner in WW conditions in both the F_1_ and F_2_ generations, while in MWDS and SWDS irrigation regimes, the P_6_ parent had the largest positive GCA value for TLY in both the F_1_ and F_2_ generations (Table 3).

**Table 3:** General combining ability-effects of parents in the F_1_ and F_2_ generations under different irrigation regimes.

F_1_ generation										
IR	P	DTF	DTEOF	DTR	UNPP	FUNPP	FNPP	TFW	FAC	TLY
**WW**	P_1_	2.44**	3.25**	1.44**	3.77**	6.37**	209.22**	0.41**	0.32^ns^	-0.17**
	P_2_	5.15**	3.58**	3.78**	4.49**	-8.45**	-15.01^ns^	-0.85**	-1.01**	0.21**
	P_3_	1.99**	1.58**	0.99**	-2.11^ns^	-7.57**	-160.43**	-0.78**	-1.64**	-0.20**
	P_4_	0.78*	2.29**	1.40**	7.28**	22.78**	223.24**	-0.12^ns^	-0.60*	0.31**
	P_5_	4.86**	3.63**	3.57**	-2.10^ns^	-4.93**	-139.64**	-0.22*	0.07^ns^	0.06^ns^
	P_6_	-15.22**	-14.33**	-11.18**	-11.33**	-8.20**	-117.37**	1.56**	2.86**	-0.21**
**MWDS**	P_1_	4.06**	3.86**	3.24**	1.58^ns^	-0.18^ns^	-37.68**	-0.73**	-0.69*	-0.19**
	P_2_	5.01**	4.78**	4.07**	-2.57^ns^	-0.43^ns^	-82.90**	-0.78**	-1.74**	-0.28**
	P_3_	3.39**	3.15**	2.15**	-3.62*	-0.91**	2.28^ns^	-0.61**	-1.19**	-0.17**
	P_4_	-0.69**	-1.97**	-1.81**	4.79**	1.82**	184.93**	-0.01^ns^	-0.44^ns^	0.35**
	P_5_	4.31**	3.57**	3.78**	0.86^ns^	-1.55**	-30.42**	-0.86**	0.06^ns^	-0.24**
	P_6_	-16.07**	-13.39**	-11.43**	-1.04^ns^	1.26**	-36.20**	2.99**	4.01**	0.53**
**SWDS**	P_1_	3.69**	2.54**	1.79**	2.80**	-0.51^ns^	-6.29^ns^	-0.71**	0.61*	-0.06^ns^
	P_2_	5.53**	4.21**	4.00**	-1.15*	-0.98**	-73.71**	-0.82**	-1.35**	-0.14**
	P_3_	2.69**	1.79**	1.33**	-1.41**	-0.82**	-22.13*	-0.65**	-0.97**	-0.06^ns^
	P_4_	1.86**	-0.04^ns^	-0.29^ns^	1.12*	1.52**	160.93**	-0.29**	-0.18^ns^	0.11**
	P_5_	4.44**	3.25**	2.58**	-0.47^ns^	-0.53^ns^	-65.67**	-0.38**	-1.39**	-0.14**
	P_6_	-18.22**	-11.75**	-9.42**	-0.89^ns^	1.33**	6.86^ns^	2.84**	3.28**	0.29**
**F**_**2**_ **generation**										
**WW**	P_1_	2.53**	3.33**	1.83**	4.01**	5.63**	163.57**	0.38**	0.05^ns^	-0.14**
	P_2_	5.57**	3.67**	4.17**	3.79**	-6.70**	-28.85^ns^	-0.77**	-0.66*	0.12*
	P_3_	2.94**	1.88**	0.83**	-2.10^ns^	-8.51**	-128.69**	-0.65**	-1.13**	-0.10*
	P_4_	0.90^ns^	2.46**	1.63**	6.55**	22.32**	212.54**	-0.06^ns^	-0.58*	0.16**
	P_5_	4.24**	3.67**	3.92**	-1.05^ns^	-4.23**	-107.83**	-0.32**	0.27^ns^	0.06^ns^
	P_6_	-16.18**	-15.00**	-12.38**	-11.20**	-8.51**	-110.74**	1.41**	2.06**	-0.09^ns^
**MWDS**	P_1_	4.29**	4.00**	3.17**	2.15^ns^	-0.27^ns^	-13.27^ns^	-0.64**	-0.52*	-0.13*
	P_2_	5.08**	4.54**	3.63**	-2.28^ns^	-0.51*	-75.36**	-0.75**	-2.26**	-0.26**
	P_3_	3.33**	3.13**	2.25**	-2.53^ns^	-0.84**	7.26^ns^	-0.62**	-0.98**	-0.14*
	P_4_	-0.58*	-1.63**	-1.58**	4.48**	1.71**	169.07**	0.06^ns^	-0.32^ns^	0.33**
	P_5_	4.13**	4.00**	3.92**	-0.22^ns^	-1.31**	-42.63**	-0.78**	-0.21^ns^	-0.23**
	P_6_	-16.25**	-14.04**	-11.38**	-1.60^ns^	1.21**	-45.07**	2.73**	4.28**	0.43**
**SWDS**	P_1_	4.13**	3.13**	1.71**	2.52**	-0.27^ns^	-9.06^ns^	-0.51**	0.62*	-0.05 ^ns^
	P_2_	5.21**	4.33**	3.92**	-1.28**	-0.96**	-68.59**	-0.73**	-1.23**	-0.11**
	P_3_	2.71**	1.79**	0.75*	-1.01**	-0.62**	-18.53*	-0.53**	-0.89**	-0.06^ns^
	P_4_	1.96**	-0.21^ns^	-0.25^ns^	1.04**	1.20**	152.83**	-0.39**	-0.22^ns^	0.09*
	P_5_	4.00**	3.25**	2.25**	-0.31^ns^	-0.47**	-59.39**	-0.50**	-1.16**	-0.11**
	P_6_	-18.00**	-12.29**	-8.38**	-0.95**	1.12**	2.74^ns^	2.66**	2.88**	0.25**

**, * and ^ns^ indicate significance at the 1% and 5% level of probability and not significant, respectively. Irrigation regime (IR), parents (P), well-watered (WW), mild water-deficit stress (MWDS), severe water-deficit stress (SWDS), days to flowering (DTF), days to the end of flowering (DTEOF), days to ripening (DTR), umbel number per plant (UNPP), fertile umbel number per plant (FUNPP), fruit number per plant (FNPP), thousand fruit weight (TFW), fatty acid content (FAC), total lipid yield (TLY).

Results of SCA analysis for DTF, DTEOF and DTR indicated that the progenies of P_6_ (H_1_×_6_, H_2_×_6_, H_3_×_6_, H_4_×_6_ and H_5_×_6_) displayed negative significant SCA-effects in both the F_1_ and F_2_ generations in all irrigation regimes (Tables 4 and 5). In WW conditions, the crosses of H_1_×_6_ and H_4_×_6_ had the largest positive significant SCA values for UNPP in both the F_1_ and F_2_ generations. Under MWDS conditions, the SCA value for UNPP was not significant. Under SWDS conditions, the cross of H_1_×_6_ had the largest positive significant SCA value for UNPP in the F_1_ generation, whereas, in the F_2_ generation, the population of H_3_×_6_ had the largest positive significant SCA value for this trait (Tables 4 and 5).

**Table 4:** Specific combining ability-effects in the F_1_ generation under different irrigation regimes.

IR	F_1_s	DTF	DTEOF	DTR	UNPP	FUNPP	FNPP	TFW	FAC	TLY
**WW**	H_1_×_2_	1.39^ns^	1.64**	0.19^ns^	7.61*	1.21^ns^	172.84**	-0.39^ns^	1.77**	-0.80**
	H_1_×_3_	1.22^ns^	-0.02^ns^	0.65^ns^	-6.26^ns^	5.06^ns^	60.59^ns^	0.48^ns^	-0.93^ns^	0.63**
	H_1_×_4_	0.10^ns^	1.27*	-0.43^ns^	2.39^ns^	9.59**	-51.48^ns^	0.20^ns^	4.69**	1.18**
	H_1_×_5_	0.68^ns^	0.93^ns^	1.07^ns^	-0.03^ns^	-2.41^ns^	76.86^ns^	-0.17^ns^	-2.64**	0.09^ns^
	H_1_×_6_	-4.90**	-5.11**	-1.18*	13.29**	5.89^ns^	200.96**	1.02**	2.90**	0.31^ns^
	H_2_×_3_	0.51^ns^	-0.02^ns^	-0.68^ns^	2.82^ns^	-5.35^ns^	-16.84^ns^	-0.41^ns^	0.07^ns^	0.29^ns^
	H_2_×_4_	1.05^ns^	1.27*	-0.10^ns^	-1.13^ns^	-5.93^ns^	-13.15^ns^	-0.77**	0.02^ns^	1.01**
	H_2_×_5_	-0.36^ns^	-0.40^ns^	1.07^ns^	0.11^ns^	-7.26**	61.73^ns^	0.62*	3.36**	0.44*
	H_2_×_6_	-2.61**	-3.11**	0.48^ns^	-6.50^ns^	-3.22^ns^	-110.44^ns^	1.14**	0.57^ns^	0.61**
	H_3_×_4_	-0.11^ns^	0.27^ns^	2.69**	6.37^ns^	16.02**	99.81^ns^	-0.24^ns^	-2.02*	0.01^ns^
	H_3_×_5_	0.80^ns^	0.60^ns^	1.52**	-0.69^ns^	-6.91*	-277.58**	-0.25^ns^	1.65*	-0.20^ns^
	H_3_×_6_	-2.45**	-1.44*	0.94^ns^	-1.03^ns^	-9.47**	-6.26^ns^	0.97**	3.19**	0.50**
	H_4_×_5_	1.68*	0.56^ns^	-0.56^ns^	0.99^ns^	15.05**	217.25**	0.19^ns^	2.27**	0.66**
	H_4_×_6_	-2.24**	-3.15**	1.86**	15.49**	9.19**	117.04*	0.58*	3.15**	0.04^ns^
	H_5_×_6_	-3.32**	-2.15**	-2.31**	-5.64^ns^	-4.71^ns^	-52.25^ns^	0.69**	-1.18^ns^	0.41*
**MWDS**	H_1_×_2_	1.76**	2.49**	1.25*	1.40^ns^	-0.90^ns^	-113.00**	-0.08^ns^	3.83**	-0.06^ns^
	H_1_×_3_	2.05**	3.78**	2.17**	-4.85^ns^	-0.95^ns^	-179.89**	-0.44^ns^	-1.71*	-0.19^ns^
	H_1_×_4_	-0.20^ns^	-2.10**	-1.88**	-2.96^ns^	0.01^ns^	89.33**	-0.76*	-0.13^ns^	0.46**
	H_1_×_5_	3.13**	-0.30^ns^	1.88**	-4.93^ns^	-0.12^ns^	-17.55^ns^	-0.10^ns^	-0.63^ns^	-0.20^ns^
	H_1_×_6_	-6.16**	-3.35**	-4.25**	4.64^ns^	2.01**	97.23**	1.25**	3.08**	0.41**
	H_2_×_3_	4.09**	4.20**	2.33**	-6.62^ns^	-0.87^ns^	-41.18*	-0.24^ns^	-0.01^ns^	-0.05^ns^
	H_2_×_4_	-2.16**	-4.68**	-4.38**	1.47^ns^	0.76^ns^	106.02**	-0.06^ns^	0.24^ns^	0.39**
	H_2_×_5_	0.51^ns^	4.78**	3.38**	-4.77^ns^	0.10^ns^	-17.13^ns^	-0.3^2^^ns^	5.08**	-0.01^ns^
	H_2_×_6_	-6.45**	-5.93**	-3.42**	5.07^ns^	0.62^ns^	77.30**	1.16**	-2.21**	0.19^ns^
	H_3_×_4_	-3.20**	-4.72**	-2.13**	-0.12^ns^	0.01^ns^	11.07^ns^	0.40^ns^	-0.96^ns^	0.01^ns^
	H_3_×_5_	2.80**	2.07**	2.96**	0.14^ns^	-0.38^ns^	-38.61*	-0.08^ns^	1.54*	0.00^ns^
	H_3_×_6_	-3.83**	-4.30**	-3.17**	5.95^ns^	0.77^ns^	62.35**	0.88*	2.91**	0.54**
	H_4_×_5_	-2.45**	-4.14**	-7.08**	-1.50^ns^	1.38^ns^	37.44*	-0.01^ns^	1.12^ns^	0.55**
	H_4_×_6_	-1.74**	-0.51^ns^	-0.21^ns^	2.57^ns^	0.67^ns^	-120.31**	0.89*	2.49**	0.74**
	H_5_×_6_	-5.08**	-4.39**	-4.46**	5.90^ns^	0.78^ns^	64.44**	0.88*	-3.67**	0.01^ns^
**SWDS**	H_1_×_2_	3.24**	1.49**	1.68**	-0.20^ns^	-0.20^ns^	4.65^ns^	-0.46*	1.91**	-0.07^ns^
	H_1_×_3_	2.74**	3.57**	1.35*	-1.08^ns^	-0.25^ns^	10.32^ns^	0.01^ns^	-0.13^ns^	-0.12^ns^
	H_1_×_4_	1.24^ns^	-0.93^ns^	2.31**	2.73^ns^	0.27^ns^	73.51**	0.24^ns^	3.08**	0.13^ns^
	H_1_×_5_	3.32**	4.11**	4.10**	-3.28*	-0.71^ns^	-57.45*	-0.17^ns^	-2.05**	-0.17^ns^
	H_1_×_6_	-6.68**	-2.55**	-3.23**	3.07*	1.69*	50.02^ns^	0.64**	1.95**	0.43**
	H_2_×_3_	2.90**	4.24**	4.14**	-0.36^ns^	-0.55^ns^	-20.22^ns^	-0.75**	-1.17^ns^	0.06^ns^
	H_2_×_4_	0.74^ns^	-0.60^ns^	0.43^ns^	0.35^ns^	1.31^ns^	86.91**	0.12^ns^	0.70^ns^	0.18^ns^
	H_2_×_5_	3.15**	1.78**	3.89**	1.47^ns^	-0.34^ns^	-35.03^ns^	0.34^ns^	2.91**	-0.11^ns^
	H_2_×_6_	-5.51**	-5.89**	-3.77**	0.59^ns^	1.23^ns^	67.52**	1.65**	3.24**	0.12^ns^
	H_3_×_4_	0.90^ns^	0.15^ns^	3.10**	0.67^ns^	0.88^ns^	57.22*	0.15^ns^	-1.01^ns^	0.10^ns^
	H_3_×_5_	1.65*	-0.80^ns^	3.56**	-0.73^ns^	-0.47^ns^	5.79^ns^	-0.16^ns^	1.87**	0.01^ns^
	H_3_×_6_	-3.68**	-2.47**	-1.77**	1.62^ns^	1.40^ns^	43.43^ns^	0.79**	2.20**	0.21^ns^
	H_4_×_5_	2.15**	0.70^ns^	-1.15*	-0.33^ns^	1.33^ns^	105.41**	-0.06^ns^	1.74*	0.25*
	H_4_×_6_	-3.85**	-1.30*	-0.82^ns^	2.42^ns^	1.40^ns^	-4.69^ns^	1.22**	2.41**	0.18^ns^
	H_5_×_6_	-5.43**	-4.60**	-3.69**	2.35^ns^	1.05^ns^	88.15**	0.98**	-2.38**	0.19^ns^

**, * and ^ns^ indicate significance at the 1% and 5% level of probability and not significant, respectively. Irrigation regime (IR), well-watered (WW), mild water-deficit stress (MWDS), severe water-deficit stress (SWDS), days to flowering (DTF), days to the end of flowering (DTEOF), days to ripening (DTR), umbel number per plant (UNPP), fertile umbel number per plant (FUNPP), fruit number per plant (FNPP), thousand fruit weight (TFW), fatty acid content (FAC), total lipid yield (TLY).

**Table 5:** Specific combining ability-effects of the F_2_ generation under different irrigation regimes.

IR	F_1_s	DTF	DTEOF	DTR	UNPP	FUNPP	FNPP	TFW	FAC	TLY
**WW**	H_1_×_2_	-1.40^ns^	1.95**	1.33**	2.69^ns^	3.48^ns^	59.66^ns^	-0.43^ns^	1.37^ns^	-0.49**
	H_1_×_3_	0.89^ns^	0.74^ns^	0.00^ns^	-0.51^ns^	2.96^ns^	47.42^ns^	0.46^ns^	-0.56^ns^	0.19^ns^
	H_1_×_4_	0.27^ns^	-0.17^ns^	0.54^ns^	0.28^ns^	6.76*	-26.76^ns^	0.23^ns^	1.96**	0.42**
	H_1_×_5_	0.60^ns^	0.62^ns^	0.25^ns^	-0.45^ns^	0.13^ns^	38.12^ns^	-0.46^ns^	-1.86*	-0.17^ns^
	H_1_×_6_	-2.32*	-6.38**	-1.46**	14.42**	3.93^ns^	135.93*	0.63*	-0.66^ns^	0.25^ns^
	H_2_×_3_	2.85*	-1.26^ns^	1.00*	2.25^ns^	-5.30^ns^	-8.04^ns^	-0.03^ns^	-0.28^ns^	-0.07^ns^
	H_2_×_4_	0.56^ns^	0.83^ns^	0.21^ns^	2.47^ns^	1.03^ns^	-21.12^ns^	-0.78**	0.12^ns^	0.32*
	H_2_×_5_	2.23^ns^	-0.38^ns^	-0.42^ns^	-1.63^ns^	-5.40^ns^	70.52^ns^	0.25^ns^	1.65*	-0.08^ns^
	H_2_×_6_	-3.36**	-3.71**	-0.79^ns^	-7.24*	-6.50*	-85.05^ns^	0.90**	-0.15^ns^	0.15^ns^
	H_3_×_4_	2.85*	0.62^ns^	2.21**	4.27^ns^	10.63**	77.09^ns^	-0.61*	-1.70*	-0.16^ns^
	H_3_×_5_	-0.48^ns^	0.41^ns^	1.58**	-3.86^ns^	-4.46^ns^	-122.20*	-0.41^ns^	1.17^ns^	0.03^ns^
	H_3_×_6_	-3.07**	-2.26**	-1.46**	-2.44^ns^	-7.42**	-30.39^ns^	1.07**	0.86^ns^	0.28*
	H_4_×_5_	0.89^ns^	0.83^ns^	1.13*	3.06^ns^	12.18**	143.20*	0.41^ns^	0.63^ns^	0.17^ns^
	H_4_×_6_	-4.36**	-3.51**	-0.92^ns^	9.57**	12.34**	131.48*	0.36^ns^	2.71**	0.22^ns^
	H_5_×_6_	-7.02**	-4.05**	-1.54**	0.26^ns^	-5.06^ns^	0.81^ns^	0.27^ns^	-1.81*	0.13^ns^
**MWDS**	H_1_×_2_	1.24*	2.60**	1.40*	-2.12^ns^	-0.61^ns^	8.69^ns^	-0.03^ns^	1.74*	-0.05^ns^
	H_1_×_3_	2.66**	3.35**	1.44*	-3.96^ns^	-1.20*	-85.61**	-0.09^ns^	-1.58*	-0.16^ns^
	H_1_×_4_	0.24^ns^	-2.57**	-2.06**	1.04^ns^	0.10^ns^	45.52*	-0.47^ns^	-0.67^ns^	0.30^ns^
	H_1_×_5_	1.87**	1.14*	1.11^ns^	-2.70^ns^	0.16^ns^	-60.84**	-0.39^ns^	-0.46^ns^	-0.14^ns^
	H_1_×_6_	-5.42**	-4.15**	-4.27**	4.39^ns^	1.02^ns^	68.71**	1.01*	4.45**	0.40**
	H_2_×_3_	3.54**	3.81**	2.32**	-0.26^ns^	-0.64^ns^	-59.14**	-0.28^ns^	0.51^ns^	0.03^ns^
	H_2_×_4_	-2.88**	-4.77**	-5.85**	-1.65^ns^	0.42^ns^	67.96**	0.35^ns^	0.32^ns^	0.15^ns^
	H_2_×_5_	2.41**	4.27**	2.32**	-2.92^ns^	-0.36^ns^	-24.65^ns^	-0.42^ns^	3.58**	-0.04^ns^
	H_2_×_6_	-7.21**	-6.69**	-4.06**	5.75^ns^	0.37^ns^	52.02*	0.77*	-2.98**	0.06^ns^
	H_3_×_4_	-2.46**	-4.36**	-1.81**	1.42^ns^	-0.17^ns^	-20.99^ns^	0.00^ns^	-0.79^ns^	0.01^ns^
	H_3_×_5_	2.49**	2.68**	3.02**	-3.55^ns^	0.16^ns^	-63.27**	-0.12^ns^	0.97^ns^	0.02^ns^
	H_3_×_6_	-5.46**	-5.27**	-3.68**	6.28^ns^	0.49^ns^	65.35**	0.78*	1.82**	0.35*
	H_4_×_5_	-3.92**	-3.57**	-6.14**	-3.65^ns^	0.90^ns^	69.70**	0.40^ns^	0.32^ns^	0.23^ns^
	H_4_×_6_	-1.21*	-0.19^ns^	-0.18^ns^	2.16^ns^	0.95^ns^	-99.36**	0.26^ns^	2.41**	0.79**
	H_5_×_6_	-5.59**	-5.48**	-4.35**	4.41^ns^	1.63**	61.51**	0.99**	-3.70**	-0.03^ns^
**SWDS**	H_1_×_2_	3.24**	2.88**	1.52^ns^	-2.04*	-0.11^ns^	-6.64^ns^	-0.34^ns^	1.68*	-0.09^ns^
	H_1_×_3_	1.74*	3.08**	1.68^ns^	-1.29^ns^	-0.04^ns^	-6.52^ns^	0.08^ns^	-0.01^ns^	-0.08^ns^
	H_1_×_4_	4.49**	2.75**	2.35**	0.97^ns^	0.30^ns^	46.00*	-0.01^ns^	2.11**	0.10^ns^
	H_1_×_5_	1.11^ns^	2.96**	2.52**	-1.76^ns^	-0.62^ns^	-22.45^ns^	0.04^ns^	-1.85*	-0.14^ns^
	H_1_×_6_	-7.22**	-3.83**	-3.52**	1.45^ns^	1.30**	45.98*	1.15**	0.84^ns^	0.12^ns^
	H_2_×_3_	2.65**	4.54**	3.14**	-1.61^ns^	-0.15^ns^	-0.53^ns^	0.08^ns^	-0.85^ns^	0.06^ns^
	H_2_×_4_	0.40^ns^	-0.13^ns^	0.48^ns^	0.78^ns^	1.01*	84.85**	0.37^ns^	1.01^ns^	0.17^ns^
	H_2_×_5_	0.36^ns^	0.75^ns^	-0.02^ns^	-0.16^ns^	-1.09*	-29.80^ns^	0.05^ns^	1.85*	-0.11^ns^
	H_2_×_6_	-5.64**	-6.71**	-0.40^ns^	1.55^ns^	0.96*	62.85**	0.95**	2.35**	0.14^ns^
	H_3_×_4_	-1.43^ns^	-1.92**	0.64^ns^	1.38^ns^	0.64^ns^	59.64**	-0.20^ns^	-1.52^ns^	0.04^ns^
	H_3_×_5_	4.20**	1.96**	3.48**	-1.52^ns^	-0.38^ns^	3.06^ns^	-0.20^ns^	1.77*	0.03^ns^
	H_3_×_6_	-4.80**	-3.17**	-2.23**	1.99*	0.86^ns^	41.73^ns^	0.62**	0.69^ns^	0.10^ns^
	H_4_×_5_	-0.05^ns^	-1.38*	0.81^ns^	-0.82^ns^	0.73^ns^	92.83**	-0.16^ns^	1.20^ns^	0.21^ns^
	H_4_×_6_	-4.05**	-2.17**	-1.57^ns^	0.42^ns^	0.34^ns^	-10.94^ns^	1.12**	1.94*	0.33**
	H_5_×_6_	-4.76**	-3.29**	-2.73**	1.64^ns^	1.55**	74.78**	0.53*	-2.00*	0.15^ns^

**, * and ^ns^ indicate significance at the 1% and 5% level of probability and not significant, respectively. Irrigation regime (IR), well-watered (WW), mild water-deficit stress (MWDS), severe water-deficit stress (SWDS), days to flowering (DTF), days to end the of flowering (DTEOF), days to ripening (DTR), umbel number per plant (UNPP), fertile umbel number per plant (FUNPP), fruit number per plant (FNPP), thousand fruit weight (TFW), fatty acid content (FAC), total lipid yield (TLY).

Under WW conditions, the progenies of the P_4_ parent (H_1_×_4_, H_3_×_4_, H_4_×_5_ and H_4_×_6_) had the largest positive significant SCA values for FUNPP in both the F_1_ and F_2_ generations. In MWDS and SWDS conditions, the crosses of H_1_×_6_ and H_5_×_6_ had the largest positive significant SCA values for FUNPP in both the F_1_ and F_2_ generations (Tables 4 and 5).

In case of FNPP, the cross of H_4_×_5_ had the largest positive significant SCA value in WW and SWDS irrigation regimes in both the F_1_ and F_2_ generations. In MWDS conditions, the crosses of H_2_×_4_ and H_4_×_5_ had the positive significant SCA values for FNPP in both the F_1_ and F_2_ generations. For TFW, the progenies of the P_6_ parent (H_1_×_6_, H_2_×_6_, H_3_×_6_, H_4_×_6_ and H_5_×_6_) had positive significant SCA values in all irrigation regimes in both the F_1_ and F_2_ generations (Tables 4 and 5).

Under WW conditions, the crosses of H_1_×_4_, H_1_×_6_, H_3_×_6_, H_4_×_5_ and H_4_×_6_ had positive significant SCA values for FAC in the F_1_ generation whereas, in the F_2_ generation, the populations of H_1_×_4_ and H_4_×_6_ had the positive significant SCA values. In MWDS conditions, the crosses of H_1_×_6_, H_3_×_6_ and H_4_×_6_ had the positive significant SCA values for FAC in both the F_1_ and F_2_ generations. Also, in SWDS conditions, the crosses of H_1_×_6_ and H_4_×_5_ had a positive significant SCA value for FAC in the F_1_ generation and the population of H_4_×_6_ had a positive significant SCA value in the F_2_ generation (Tables 4 and 5).

Under WW conditions, the crosses of H_1_×_4_ and H_2_×_4_ had the largest positive significant SCA values for TLY in both the F_1_ and F_2_ generations. Under MWDS conditions, the crosses of H_1_×_6_, H_3_×_6_ and H_4_×_6_ had the largest positive significant SCA values for TLY in both the F_1_ and F_2_ generations. Also, under SWDS conditions, the crosses of H_1_×_6_ and H_4_×_5_ had the largest positive significant SCA values for TLY in the F_1_ generation and the population of H_4_×_6_ had the largest positive significant SCA value in the F_2_ generation (Tables 4 and 5).

### Genetic correlation of total lipid yield with phenological and morphological traits

Genetic correlation analysis under WW conditions showed that there were positive correlations between total lipid yield and all of the phenological and yield components traits (S4 Table). In MWDS and SWDS conditions, total lipid yield was significantly and positively correlated with yield components while total lipid yield had the significant negative correlation with phenological traits (S4 Table).

## Discussion

Iran has special a geographical location with high genetic diversity for coriander, as well as many other crops. It is a promising place to find and gather new genetic resources for coriander anda high genetic diversity was previously reported for drought stress tolerance for Iranian coriander genotypes [35]. Using drought-tolerant and high-yielding coriander genotypes as the parents in crossing programs can significantly increase the efficiency of coriander breeding schemes for developing high-yielding coriander genotypes for arid and semi-arid areas. In this study, a large genetic variation was observed for phonological and yield components, total lipid yield and fatty acid content among the parental genotypes, F_1_ hybrids and F_2_ populations. This indicates the existence of an excellent potential to study coriander genetics relative to crop improvement.

Results showed that flowering and maturity times were decreased in MWDS and SWDS irrigation regimes. In agreement with our results, Gales and Wilson [43] with studies in winter wheat, Bannayan et al. [20] in isabgol and black cumin and Alinian and Razmjoo [23] in cumin (*Cuminum Cyminum* L.) reported that water-deficit stress induced a reduction in the time to maturity. This effect is influenced by various factors including the level and duration of the stress, the genotype and the maturity time under non-stress conditions. Reduced time to maturity is known as a water-deficit stress avoidance mechanism in plants [43].

Yield components’ measurements were higher under WW condition than with MWDS and SWDS irrigation regimes. Reduction in yield components may be due to lower availability of nutrients along with reduced photosynthesis and reduced translocation of photosynthesis products from source to sink area under drought stress [44]. The preferential allocation of biomass to the root growth has been associated with yield reduction in coriander under water stress conditions [26]. Similarly, Alinian and Razmjoo [23] reported that number of umbels per plant, number of seeds per umbel and 1000 seeds weight were reduced under water-deficit stress in cumin accessions.

The highest fatty acid content and total lipid yield values observed under WW conditions, conversely the lowest fatty acid content and total lipid yield values were obtained in SWDS conditions in both the F_1_ hybrids and the F_2_ populations. Similar results were reported by Singh and Ramesh [45] in rosemary, Zehtab-Salmasi et al. [19] in dill (*Anethum graveolens* L.), Hamrouni et al. [13] in safflower, Bettaieb et al. [11] in *Salvia officinalis* L. and Bettaieb et al. [14] in cumin (*Cuminum cyminum* L.). Those studies observed that fatty acid content and total lipid yield were significantly decreased by water-deficit stress. Reduction in total lipid yield under water-deficit stress could also be due to the reduction in days to flowering and maturity and some of the yield components for plants.

Narrow-sense heritability and GCA/SCA ratio values suggest that additive genetic effects were predominant in controlling phenological and yield components traits in all irrigation regimes in both the F_1_ and F_2_ generations. Therefore, breeding methods based on selection can be effective in the F_2_ generations for improvement of these traits. Similar to our findings, Amiri-Oghan et al. [25] observed a high heritability for days to flowering and days to maturity in oilseed rape (*Brasica napus* L.). FAC and TLY In coriander were predominantly affected by non-additive gene action in the F_1_ generation while additive gene action predominated in the F_2_ generation under MWDS and SWDS conditions. Therefore, breeding methods based on selection in the F_2_ and later generations will likely be effective to improve FAC and TLY in MWDS and SWDS conditions. The results of narrow-sense heritability and the GCA/SCA ratio for TLY in WW indicate that non-additive type of gene action were predominant in both the F_1_ and F_2_ generations. Therefore, for improvement of TLY under WW conditions, selection should be deferred to the later generations of segregation in which non-additive genetic effects have been reduced or fixed.

Blum [46] reported that indirect selection for yield components and other traits that have high heritability and which are strongly correlated with economical yield could be more efficient than direct selection for yield. In this study phenological and TFW traits had higher heritability estimates than total lipid yield. These traits also had a significant genetic correlation with total lipid yield. Thus, selection for DTF, DTEOF, DTR and TFW traits may be effective criteria for improvement of total lipid yield, especially under water stress conditions. The significant and negative genetic correlations between total lipid yield and phenological traits in MWDS and SWDS conditions suggest that simultaneous improvement of earliness to cope with drought stress and total lipid yield can be achieved in coriander. Flowering is the most critical stage that influences the yield of coriander. The time of flower initiation can have a strong influence on the number of flowers, umbel number per plant, fertile umbel number per plant and fruit number per plant. The development of early ripening coriander genotypes is important to avoid abiotic stresses, particularly drought and high temperatures at the end of the growing season in arid and semi-arid environments. Such conditions have been observed in some areas of Iran and coriander production has been restricted by the adverse effects of the terminal heat and drought stress which cause a reduction in the number of successfully pollinated flowers. Overall, the importance of phenological traits and the use of early genotypes as donor parents should be considered in coriander breeding programs to improve total lipid yield.

Achievement in breeding programs depends on the careful choice of parents. The selection of parents for hybridization programs should be based on their genetic value. High GCA values of the parents are mainly due to the additive genetic effects [37] that are heritable in the segregating generations. Therefore, the selection of parents for hybridization should be based on their GCA-effects which reflect on their potential to produce superior segregates in the F_2_ and later generations. The GCA-effects of the six parents on the traits measured in both the F_1_ and F_2_ generations showed that the P_4_ appeared as the best general combiner for UNPP, FUNPP and FNPP. P_6_ was the best general combiner for DTF, DTEOF, DTR, TFW, FAC and TLY. These parents could be used to develop early flowering and early maturing types coupled with high total lipid yield genotypes in advanced segregating generations. The offspring of the P_4_ and P_6_ parents had high SCA values for FAC and TLY. They also exhibited significant SCA values for other phenological and yield components traits in both F_1_ and F_2_ generations. Results of the GCA and SCA analysis indicate that many of crosses which showed significant SCA-effects also had high GCA values for all traits.

## Conclusion

Large genetic variability for phenological, yield components, total lipid yield and fatty acid content indicate a high potential of the studied germplasm for genetic improvement in coriander. The results indicated that water-deficit stress decreased DTF, DTEOF, DTR, UNPP, FUNPP, FNPP, TFW, FAC and TLY in both F_1_ hybrids and F_2_ generations. The high narrow-sense heritability and GCA/SCA ratio for phenological traits indicates that these traits are mainly governed by additive genetic effects and suggest that these traits can be used as reliable and heritable selection criteria under drought stress. These traits were also correlated with total lipid yield and could be used as suitable surrogate selection criteria to enhance total lipid yield and to identify superior genotypes for drought stress conditions. Based on their general combining ability, the P_4_ and P_6_ parents can be used as promising parents for hybridization and selection of genotypes with high total lipid yield coupled with early ripening in advanced generations of segregation.

## Supporting information

S1 Data File Supporting information file for the paper, data file used in this manuscript’s analyses. (XLS)

S1 Table Soil properties of different layers of the experimental field. FC, soil moisture at field capacity.
(DOC)

S2 Table Combined analysis of variance for traits in the F_1_ and F_2_ progenies and their parents under drought environment. **,* and ^ns^ indicate significance at the 1% and 5% level of probability and not significant, respectively. Environment (E), replication (R), genotype (G), general combining ability (GCA), specific combining ability (SCA), days to flowering (DTF), days to the end of flowering (DTEOF), days to ripening (DTR), umbel number per plant (UNPP), fertile umbel number per plant (FUNPP), fruit number per plant (FNPP), thousand fruit weight (TFW), fatty acid content (FAC), total lipid yield (TLY).
(DOC)

S3 Table The mean of traits in coriander under different irrigation regimes in the F_1_ and F_2_ generations. In each column, the values with the same letters do not differ significantly. Well-watered (WW), mild water-deficit stress (MWDS), severe water-deficit stress (SWDS), days to flowering (DTF), days to the end of flowering (DTEOF), days to ripening (DTR), umbel number per plant (UNPP), fertile umbel number per plant (FUNPP), fruit number per plant (FNPP), thousand fruit weight (TFW), fatty acid content (FAC), total lipid yield (TLY).
(DOC)

S4 Table Genetic correlation coefficients and their standard error (SE) between total lipid yield and other traits under different irrigation regimes. Well-watered (WW), mild water-deficit stress (MWDS), severe water-deficit stress (SWDS), days to flowering (DTF), days to the end of flowering (DTEOF), days to ripening (DTR), umbel number per plant (UNPP), fertile umbel number per plant (FUNPP), fruit number per plant (FNPP), thousand fruit weight (TFW), total lipid yield (TLY). ** indicates statistical significance at the 1% level of probability.
(DOC)
