# Supplementary material for: Genetic combining ability of coriander genotypes for agronomic and phytochemical traits in response to contrasting irrigation regimes
Source: PLoS One. 2018 Jun 28;13(6):e0199630. doi: 10.1371/journal.pone.0199630 (PMC6023167; doi:10.1371/journal.pone.0199630)
Supplement: S1 Table — FC, soil moisture at field capacity. (DOC) [file pone.0199630.s002.doc]

**S1 Table. Soil properties of different layers of the experimental field.**

| **Soil depth (cm)** | **Sand (%)** | **Silt (%)** | **Clay (%)** | **Bulk density (g cm^−3^)** | **FC (%)** | **Organic matter (%)** | **pH** | **EC (dS m^−1^)** |
| --- | --- | --- | --- | --- | --- | --- | --- | --- |
| 0-20 | 70 | 15 | 15 | 1.2 | 16.5 | 1.61 | 7.75 | 1.3 |
| 20-40 | 68 | 18 | 14 | 1.4 | 19 | 1.45 | 7.75 | 1.28 |
| 40-60 | 66 | 18 | 16 | 1.48 | 15 | 1.09 | 7.74 | 1.26 |

FC, soil moisture at field capacity.
