# Supplementary material for: Genetic combining ability of coriander genotypes for agronomic and phytochemical traits in response to contrasting irrigation regimes
Source: PLoS One. 2018 Jun 28;13(6):e0199630. doi: 10.1371/journal.pone.0199630 (PMC6023167; doi:10.1371/journal.pone.0199630)
Supplement: S2 Table — **,* and ns indicate significance at the 1% and 5% level of probability and not significant, respectively. Environment (E), replication (R), genotype (G), general combining ability (GCA), specific combining ability (SCA), days to flowering (DTF), days to the end of flowering (DTEOF), days to ripening (DTR), umbel number per plant (UNPP), fertile umbel number per plant (FUNPP), fruit number per plant (FNPP), thousand fruit weight (TFW), fatty acid content (FAC), total lipid yield (TLY). (DOC) [file pone.0199630.s003.doc]

**S2 Table. Combined analysis of variance for traits in the F_1_ and F_2_ progenies and their parents under drought environment.**

| **F_1_ generation** | | | **Mean Squares** | | | | | | | | |
| --- | --- | --- | --- | --- | --- | --- | --- | --- | --- | --- | --- |
| **Source** | **df** | **DTF** | | **DTEOF** | **DTR** | **UNPP** | **FUNPP** | **FNPP** | **TFW** | **FAC** | **TLY** |
| E | 2 | 238.88^**^ | | 1454.12^**^ | 2392.77^**^ | 47275.40^**^ | 15767.68^**^ | 13182145.89^**^ | 43.86^**^ | 223.12^**^ | 35.08^**^ |
| R (E) | 6 | 2.76 | | 2.66 | 5.58 | 150.14 | 17.28 | 3455.14 | 0.19 | 5.02 | 0.70 |
| G | 20 | 1330.65^**^ | | 861.91^**^ | 577.16^**^ | 297.95^**^ | 495.41^**^ | 235343.76^**^ | 32.61^**^ | 102.71^**^ | 2.25^**^ |
| G × E | 40 | 15.28^**^ | | 22.78^**^ | 25.68^**^ | 130.23^**^ | 360.79^**^ | 66167.75^**^ | 1.72^**^ | 6.13^**^ | 0.60^**^ |
| GCA | 5 | 4889.64^**^ | | 3137.60^**^ | 2123.40^**^ | 753.30^**^ | 1565.86^**^ | 784368.45^**^ | 108.98^**^ | 219.99^**^ | 2.51^**^ |
| SCA | 15 | 144.32^**^ | | 103.34^**^ | 61.75^**^ | 146.17^**^ | 138.59^**^ | 52335.53^**^ | 7.16^**^ | 63.61^**^ | 2.16^**^ |
| GCA × E | 10 | 25.91^**^ | | 37.57^**^ | 26.27^**^ | 307.23^**^ | 1122.94^**^ | 168935.69^**^ | 5.70^**^ | 8.65^**^ | 1.13^**^ |
| SCA × E | 30 | 11.74^**^ | | 17.85^**^ | 25.49^**^ | 71.23^**^ | 106.74^**^ | 31911.78^**^ | 0.40^ns^ | 5.29^**^ | 0.42^**^ |
| Error | 120 | 1.83 | | 0.97 | 1.07 | 30.74 | 12.73 | 5519.60 | 0.32 | 1.98 | 0.05 |
| **F_2_ generation** | | |  | | | | | | | | |
| E | 2 | 274.70^**^ | | 1143.62^**^ | 2423.29^**^ | 47029.41^**^ | 16596.13^**^ | 12920254.93^**^ | 32.42^**^ | 111.27^**^ | 11.53^**^ |
| R (E) | 6 | 1.43 | | 3.37 | 2.71 | 120.25 | 32.45 | 4138.09 | 0.30 | 3.68 | 0.53 |
| G | 20 | 1392.28^**^ | | 963.22^**^ | 571.46^**^ | 255.21^**^ | 435.96^**^ | 192784.75^**^ | 26.33^**^ | 63.95^**^ | 0.93^**^ |
| G × E | 40 | 15.03^**^ | | 21.82^**^ | 27.01^**^ | 102.01^**^ | 329.93^**^ | 35897.20^**^ | 1.46^**^ | 7.10^**^ | 0.23^**^ |
| GCA | 5 | 5045.33^**^ | | 3425.29^**^ | 2128.82^**^ | 711.45^**^ | 1445.23^**^ | 675999.86^**^ | 92.42^**^ | 182.17^**^ | 1.64^**^ |
| SCA | 15 | 174.60^**^ | | 142.54^**^ | 52.33^**^ | 103.13^**^ | 99.54^**^ | 31713.05^**^ | 4.31^**^ | 24.54^**^ | 0.69^**^ |
| GCA × E | 10 | 18.33^**^ | | 35.05^**^ | 44.61^**^ | 254.88^**^ | 1074.80^**^ | 102588.38^**^ | 4.64^**^ | 13.54^**^ | 0.69^**^ |
| SCA × E | 30 | 13.93^**^ | | 17.41^**^ | 21.14^**^ | 51.06^**^ | 81.64^**^ | 13666.80^**^ | 0.40^ns^ | 4.95^**^ | 0.08^*^ |
| Error | 120 | 3.08 | | 1.31 | 1.73 | 26.59 | 10.07 | 5795.27 | 0.33 | 2.09 | 0.05 |

^**^ ,^*^ and ^ns^ indicate significance at the 1% and 5% level of probability and not significant, respectively . Environment (E), replication (R), genotype (G), general combining ability (GCA), specific combining ability (SCA), days to flowering (DTF), days to end of flowering (DTEOF), days to ripening (DTR), umbel number per plant (UNPP), fertile umbel number per plant (FUNPP), fruit number per plant (FNPP), thousand fruit weight (TFW), fatty acid content (FAC), total lipid yield (TLY).
