# Supplementary material for: Genetic combining ability of coriander genotypes for agronomic and phytochemical traits in response to contrasting irrigation regimes
Source: PLoS One. 2018 Jun 28;13(6):e0199630. doi: 10.1371/journal.pone.0199630 (PMC6023167; doi:10.1371/journal.pone.0199630)
Supplement: S3 Table — In each column, the values with the same letters do not differ significantly. Well-watered (WW), mild water-deficit stress (MWDS), severe water-deficit stress (SWDS), days to flowering (DTF), days to the end of flowering (DTEOF), days to ripening (DTR), umbel number per plant (UNPP), fertile umbel number per plant (FUNPP), fruit number per plant (FNPP), thousand fruit weight (TFW), fatty acid content (FAC), total lipid yield (TLY). (DOC) [file pone.0199630.s004.doc]

**S3 Table. The mean of traits under different irrigation regimes in F1 and F2 generations of coriander.**

| **Irrigation treatment** | **DTF** | | **DTEOF** | | **DTR** | | **UNPP** | | **FUNPP** | | **FNPP** | | **TFW** | | **FAC** | | **TLY** | |
| --- | --- | --- | --- | --- | --- | --- | --- | --- | --- | --- | --- | --- | --- | --- | --- | --- | --- | --- |
|  | F_1_ | F_2_ | F_1_ | F_2_ | F_1_ | F_2_ | F_1_ | F_2_ | F_1_ | F_2_ | F_1_ | F_2_ | F_1_ | F_2_ | F_1_ | F_2_ | F_1_ | F_2_ |
| WW | 75.68^a^ | 75.30^a^ | 89.52^a^ | 88.38^a^ | 99.25^a^ | 98.67^a^ | 74.70^a^ | 73.92^a^ | 36.47^a^ | 36.89^a^ | 1165.69^a^ | 1154.29^a^ | 7.77^a^ | 7.47^a^ | 20.59^a^ | 18.35^a^ | 1.88^a^ | 1.22^a^ |
| MWDS | 71.84^c^ | 71.38^c^ | 83.21^b^ | 82.86^b^ | 90.11^b^ | 89.48^b^ | 30.82^b^ | 31.36^b^ | 9.18^b^ | 9.07^b^ | 421.92^b^ | 423.27^b^ | 6.49^b^ | 6.41^b^ | 18.60^b^ | 17.76^b^ | 0.87^b^ | 0.73^b^ |
| SWDS | 73.21^b^ | 72.10^b^ | 80.10^c^ | 80.00^c^ | 87.52^c^ | 86.86^c^ | 24.35^c^ | 22.96^c^ | 8.96^b^ | 8.50^b^ | 332.47^c^ | 325.69^c^ | 6.20^c^ | 6.10^c^ | 16.83^c^ | 15.81^c^ | 0.43^c^ | 0.37^c^ |

In each column, the values with the same letters do not differ significantly. Well-watered (WW), mild water-deficit stress (MWDS), sever water-deficit stress (SWDS), days to flowering (DTF), days to end of flowering (DTEOF), days to ripening (DTR), umbel number per plant (UNPP), fertile umbel number per plant (FUNPP), fruit number per plant (FNPP), thousand fruit weight (TFW), fatty acid content (FAC), total lipid yield (TLY).
