# Supplementary material for: Genetic combining ability of coriander genotypes for agronomic and phytochemical traits in response to contrasting irrigation regimes
Source: PLoS One. 2018 Jun 28;13(6):e0199630. doi: 10.1371/journal.pone.0199630 (PMC6023167; doi:10.1371/journal.pone.0199630)
Supplement: S4 Table — Well-watered (WW), mild water-deficit stress (MWDS), severe water-deficit stress (SWDS), days to flowering (DTF), days to the end of flowering (DTEOF), days to ripening (DTR), umbel number per plant (UNPP), fertile umbel number per plant (FUNPP), fruit number per plant (FNPP), thousand fruit weight (TFW), total lipid yield (TLY). ** indicates statistical significance at the 1% level of probability. (DOC) [file pone.0199630.s005.doc]

**S4 Table. Genetic correlation coefficients and their standard error (SE) in parenthesis between fatty oil yield and other traits under irrigation regimes.**

| **Irrigation Regime** | **Estimates** | **DTF** | **DTEOF** | **DTR** | **UNPP** | **FUNPP** | **FNPP** | **TFW** |
| --- | --- | --- | --- | --- | --- | --- | --- | --- |
| WW | TLY | 0.55^**^$\pm$0.12 | 0.47^**^$\pm$0.13 | 0.45^**^$\pm$0.14 | 0.55^**^$\pm$0.17 | 0.49^**^$\pm$0.13 | 0.45^**^$\pm$0.14 | 0.73^**^$\pm$0.08 |
| MWDS | TLY | -0.69^**^$\pm$0.09 | -0.75^**^$\pm$0.08 | -0.77^**^$\pm$0.07 | 0.73^**^$\pm$0.14 | 0.98^**^$\pm$0.04 | 0.63^**^$\pm$0.11 | 0.65^**^$\pm$0.10 |
| SWDS | TLY | -0.83^**^$\pm$0.06 | -0.87^**^$\pm$0.05 | -0.82^**^$\pm$0.07 | 0.48^**^$\pm$0.17 | 0.88^**^$\pm$0.03 | 0.68^**^$\pm$0.10 | 0.86^**^$\pm$0.06 |

Well-watered (WW), mild water-deficit stress (MWDS), sever water-deficit stress (SWDS), days to flowering (DTF), days to end of flowering (DTEOF), days to ripening (DTR), umbel number per plant (UNPP), fertile umbel number per plant (FUNPP), fruit number per plant (FNPP), thousand fruit weight (TFW), total lipid yield (TLY).

** indicates statistical significance at the 1% level of probability.
